# Comparing the effects of SMS-based education with group-based education and control group on diabetes management: a randomized educational program

**DOI:** 10.1186/s12875-022-01820-w

**Published:** 2022-08-19

**Authors:** Hourvash Haghighinejad, Leila Liaghat, Fatemeh Malekpour, Peyman Jafari, Kaveh Taghipour, Mehrdad Rezaie, Parisa Jooya, Hamidreza Ghazipoor, Mani Ramzi

**Affiliations:** 1 Department of Family medicine, Hematology research center, Shiraz University of Medical Sciences, Shiraz, Iran; 2 Department of Family medicine, Shiraz University of Medical Sciences, Shiraz, Iran; 3 Department of biostatistics, Shiraz University of Medical Sciences, Shiraz, Iran; 4 Department of Family Medicine, Department of Pediatric Medicine, Neonatology Research Center, Shiraz University of Medical Sciences, Shiraz, Iran; 5 Department of hematology and oncology and stem cell transplantation, Hematology research center, Shiraz University of Medical Sciences, Shiraz, Iran

**Keywords:** Diabetes mellitus, Mobile health education, Group education, Self-management, Lifestyle modification

## Abstract

**Background:**

The high prevalence of diabetes and the importance of long-term follow-up of these patients encourage finding an inexpensive and applicable educational method to control the disease. Distance education based on mobile technology and Short message service (SMS) can be an effective way to manage this disease by eliminating time and place limitations. Due to the world’s high penetration rate, SMS is one of the best ways to transfer information and health education.

**Objective:**

This study aimed to compare the effect of SMS- and group-based education in managing diabetes type 2 and compare them with a control group.

**Method:**

A total of 168 patients with diabetes type 2 under the coverage of three family physician clinics were randomly allocated into three groups. The education was conducted in 12 one-hour sessions once a week in the group-based arm, and a daily short message was sent to the participants in the SMS group. The control group also underwent routine care at the family physician clinic. The duration of the education was 3 months. At baseline and 3 months later, fasting blood sugar (FBS), 2 hours postprandial sugar (2hppBS), and HBA1c, as well as diabetes self-management questionnaire score (DSMQ), were measured.

**Results:**

The comparison of the three groups in terms of changes in FBS (*P*-value: 0.001), 2hppBS (8 *P*-value: < 0.001) and HbA1c (*P*-value: < 0.001) were significantly different after 3 months. In pairwise analysis, 2hppBS was the only significantly different parameter between the group- and SMS-based education (*P*-value: 0.035).

**Conclusion:**

Although the effect of both educational methods via SMS or group education was better than the control group in controlling diabetes, these two methods were not statistically different. Due to spending a lot of time and money on group-based education, it is better to replace it with education by SMS.

## Introduction

Diabetes is one of the most critical health problems and the most common chronic metabolic disease in the world [1]. The number of diabetics is estimated to increase to 439 million worldwide by 2030 [2].

Diabetes type 2, a chronic and progressive disease, is a multifactorial condition caused by genetic and environmental factors [2].

People with diabetes continuously need to make the right decisions every day to maintain their blood sugar in an acceptable range. Behavioral changes are almost always necessary to maintain a healthy lifestyle and reduce the risk of long-term complications [2]. In fact, self-care is a scientifically proven and active process that reduces the risk of short-term and long-term complications of the disease and improves the quality of life [3]. Lack of a self-care program for diabetic patients is the most crucial underlying cause of death [3].

A diabetic patient must be educated about his disease to take good care of himself [4].

Complications and problems caused by diabetes significantly impact the quality of life of individuals and families, impose a high cost on the individual and the economy of the society, and account for at least 10% of total health care costs in many countries. The high prevalence of diabetes and the importance of long-term follow-up encourage health authorities to find an inexpensive and applicable education method for diabetics. Developing countries have limited resources; therefore, they need careful educational planning to control chronic diseases [5].

Education can be done in different ways, such as group-based education, brochures, mobile phones, and the Internet. Mobile health (mhealth), which is an emerging issue in the health care system, has attracted more attention in recent decades. It is a general term for using mobile technology, including phones, tablets, and trade devices, to improve public health and health care.

A systematic review of other review studies on the use of mobile technology to improve various health conditions in 2018 showed that this technology can work in many situations. This study ultimately concluded that the evidence, in this case, is still limited, and the number of high-quality studies is minimal. Likewise, most of these studies are implemented in high-income countries, with little data from low-income societies [6].

Short message service (SMS) technology as part of the mhealth strategy is one of the best ways to transfer information and health education [7]. Currently, 91.54% of the world’s population has a mobile phone [8]. The rate of mobile phone use in Iran as a developing country is 98%, and mobile users read about 90% of text messages [9]. Meanwhile, the penetration rate of smartphones in Iran is reported to be 62.9%, much less than text message usage [8]. Therefore, in the patient access process in this country, like in other developing countries, SMS can be used as the first comprehensive device.

According to research, many patients with type 2 diabetes face barriers to self-care, including economic, educational, social, psychological, and physical barriers [10]. Since it may be difficult for some people with diabetes and elderly patients to visit medical centers, distance education and follow-up by emerging educational methods based on mobile technology and SMS service could effectively control diabetes. These methods can eliminate time and place limitations in establishing a caring relationship and have a lower cost [5].

A systematic review of sixteen studies showed that SMS intervention significantly impacted the glycemic level or health behavior of diabetic patients. Finally, the authors concluded that more studies are needed to determine the frequency, time interval, and duration of intervention to be more effective [7].

On the other hand, the evidence about the superiority or inferiority of this new method over traditional group-based education is not conclusive, especially in middle- and low-income countries. Therefore, we decided to compare the effect of these two methods in the management of diabetes type 2 and compare them with a control group.

## Method

### Study setting and design

In this three-arm randomized experimental study, 168 patients with diabetes type 2 under the coverage of 3 outpatient clinics of family physicians in Shiraz were included. Shiraz is the fifth-most-populous city in Iran. These clinics affiliated to Shiraz University of Medical Sciences were located in three different areas in the town: Emam Reza and Razool Azam clinics in relatively low socioeconomic areas and Kowsar Health Center in a moderate socioeconomic region. Seven family physicians employed in the clinics agreed to cooperate in the research. Overall, 690 diabetic patients were under the coverage of these physicians.

Sample size calculation: The sample size was calculated using PASS11 software for the ANOVA test considering α = 0.05, power level: 80%, the difference between HbA1c = 1.4 and standard deviation (S.D.) based on previous studies was 1.3 [11] and 20% of missing samples.

The total calculated sample size was 168 patients, 56 for each group. Proportional sampling was done based on the number of patients covered by each center.

On arrival, the patients were examined for eligible criteria. Inclusion criteria were: age 30 to 70 years; having uncontrol type 2 diabetes; not on insulin therapy; had no other underlying disease including cardiovascular disease, kidney failure, peripheral vascular disease; and able to use a cell phone. Uncontrol diabetes defined as FBS > 130, 2 hr. PPBS> 180 or HBA1c > 7%.

Exclusion criteria included pregnant or breastfeeding women, psychiatric disorders, and micro- and macrovascular complications of diabetes.

The patients who met the eligible criteria completed the informed consent form and were interviewed and examined by a family physician resident responsible for the project. Also, if necessary, they were referred to an internal specialist who confirmed the patients’ eligibility to recruit for the research. Then, the participants completed the data collection form, including demographic information and the Diabetes Self-Management Questionnaire (DSMQ). HbA1c, 2 hours-postprandial sugar (2hppBS), and Fasting blood sugar (FBS) were requested to be measured. Patients were allocated into group-based education, education by SMS, and control groups based on the sealed envelope method when the laboratory test results were prepared. A randomization website created the randomization list with block size 6. Based on the random allocation list, sealed envelopes containing the allocation group were prepared and arranged by one of the researchers who was not involved in the education program. Sampling continued until the number of participants reached the specified sample size level. Finally, after 3 months, all participants in three groups rechecked FBS, 2hppBS, and Hb A1c and refilled the questionnaire (DSMQ) at most 1 week after intervention completion.

#### Educational program

The duration of the intervention was 3 months. Educational materials were extracted from the patient education section of the UpToDate evidence-based site. The family physician resident conducted the training. The participants in the group-based education were trained by power-point presentations for 12 one-hour sessions once a week. Also, they asked their questions for 15 minutes at the end of each session. The SMS training group received a short message daily. Same SMS messages were generally sent to all subjects. Patients were asked to respond briefly to the text message to ensure they received it. This group could also send their questions by SMS to the family medicine resident for 1 hour on Tuesdays and receive the answer. Two family medicine faculty members reviewed all training in the group- and SMS-based education. The SMS was sent by creating a group of participants and sending messages by phone. The control group also underwent routine care at the family medicine clinic, which consisted of face-to-face education about diet and physical activity by a health care worker at each visit, physician visits as demanded, and follow-up regularly at least every 3 months. The participants in 2 other groups did not get face-to-face education but also followed up and were visited by their family physicians as demanded. Nine topics of the diabetes self-management education program were used to educate both intervention groups. These items include: Describing the stages of diabetes and treatment options, Combining Nutrition Management with Lifestyle, Combining physical activity with lifestyle, Safe use of drugs to achieve a maximum therapeutic effect, Blood sugar monitor, and other parameters and their interpretation, Prevention, identification and treatment of acute complications, Prevention, identification and treatment of chronic complications, Developing Personal Strategies for Identifying Psychosocial Issues and Concerns, Developing personal strategies to promote health and behavioral change [12].

#### Outcomes measurement

HbA1c was evaluated as the primary outcome. The other outcomes were FBS, 2hrPPBS, and Diabetes Self-Management Questionnaire (DSMQ).

HbA1c, 2hppBS, and FBS were measured by the standard laboratories before allocation to the 3 study groups and 3 months later, at most 1 week after education completion.

Diabetes Self-Management Questionnaire (DSMQ), a self-administered scale, consists of 16 questions in 5 subscales: Diabetes-related nutritional behavior control, blood sugar monitoring, following medication instructions, physical activity, and doctor visits. It consists of seven positive and nine negative items and is rated using four-point Likert scoring. Negative items were scored reversely so that a higher score indicates better self-management. The sub-scales score (as well as the whole questionnaire) was the sum of items’ points divided by the maximum possible score in the sub-scale (or the entire questionnaire) multiplied by 10. The original questionnaire had good internal consistency (Cronbach’s alpha:0.84) and a mean item-total correlation of 0.46 [13]. The validity and reliability of the Persian version of this questionnaire have been proven. Cronbach’s alpha of the questionnaire was 0.72, and the internal correlation coefficient of the test-retest was above 0.72 in all subscales [14]. The questionnaire was completed before allocation to the 3 study groups and 3 months later, at most 1 week after education completion.

### Cost measurement

To compare the cost of 2 educational methods in this study, we roughly estimate the direct cost by measuring 3 items, including the average fee for sending each SMS, personal wages per hour, and the average fee a person paid for transportation from home to clinics**.** The cost of each SMS with medium length was asked from the telecommunications company. The wage of a health care worker with a bachelor’s degree was requested from the Shiraz University of Medical Science. The transportation cost was calculated by estimating the taxi fare for 8 km round trip. (Since the family doctor’s clinic should be close to the person’s home, the distance is not more than 4 km.)

#### Statistical analysis

Data were analyzed by IBM SPSS Statistics for Windows, version 26 (IBM Corp., Armonk, N.Y., USA) software. Descriptive data were expressed by frequency and graphs. To compare the data before and after intervention in each group Pair t-test was used. The ANOVA test was used to compare the raw DSMQ questionnaire scores and the changes in glycemic levels between the three groups. The changes in glycemic levels were compared between the three groups because it is essential to precisely estimate the effect of educational methods on these indices. After training, the raw DSMQ questionnaire scores were compared between the three groups to simplify the statistical analysis and interpret questionnaire results.

A Chi-square test was used to compare the frequency between the three groups. The statistical significance level was set at 0.05. The effect size was calculated by Cohen’s d. The value of effect size < 0.2, 0.2–0.49, 0.5–0.79 and > 0.8 are considered as negligible, small, moderate and large, respectively [15].

## Results

**Fig. 1:**
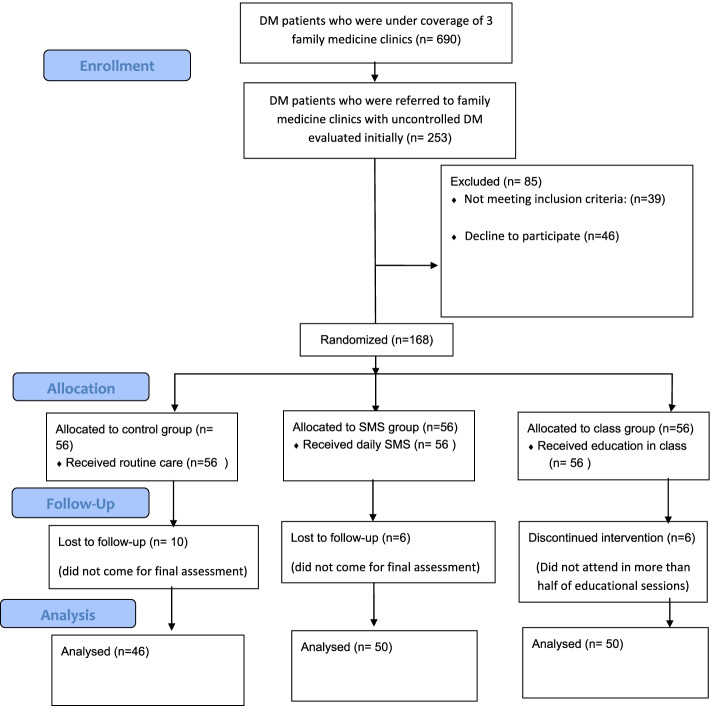
Participants’ flow chart. DM: Diabetes Mellitus

In this study, 253 people were initially examined. Finally, 168 people eligible to participate in the research and willing to cooperate were included. The recruitment flow chart is illustrated in Fig. 1. Twenty-two participants were lost to follow-up: 10 in the control group and 6 in each interventional group (Fig. 1). Finally, the information of 46 people in the control group, 50 people in the SMS group, and 50 people in the group-based education arm were statistically analyzed.

The mean age of participants was 57.5, 54, and 57.1 years in the control group, the SMS group, and the group-based training, respectively. (F (2, 142): 2.8; *P*-value: 0.06). There were no significant differences between the 3 groups regarding education, gender, and marital status. The level of FBS, 2hppBS, Hb A1c, and the Diabetes Management Questionnaire (DSMQ) score showed no statistically significant difference in the three groups at baseline (Table 1).

**Table 1:** Participants’ Characteristics in 3 groups of study

**Variables**	**Control group** **No. (%)**	**SMS group** **No. (%)**	**Group-based education** **No. (%)**	***P*-value**
**Gender**				
**Male**	23 (50)	20 (40)	16 (32)	0.199
**Female**	23 (50)	30 (60)	34 (68)	
**Education**				
**Lesser than diploma**	39 (84.8)	35 (70)	43 (86)	0.067
**Diploma or higher**	7 (15.2)	15 (30)	4 (8)	
**Marital status**				
**Single**	0	3 (6)	0	0.208
**Married**	46 (100)	47 (94)	50 (100)	
	**Control group** **Mean (S.D.)**	**SMS group** **Mean (S.D.)**	**Group-based education Mean (S.D.)**	***P*-value**
**Mean age, years**	57.5 (9)	54 (8.5)	57.1 (7.4)	0.064
**Baseline Weight, kg**	75.3 (10.8)	75.9 (15.6)	74.1 (12)	0.917
**Baseline BMI**	28 (0.6)	28.1 (0.7)	29 (1)	0.586

### Before-after comparison

After the study, the means of the three glucose indicators (FBS, 2hppBS, Hb A1c) and the score of the DSMQ were better than baseline values in the group-based education and SMS-based education (*P*-value < 0.05). The control group showed no significant changes after 3 months.

As shown in Table 2, the average FBS levels decreased after the study compared to before the intervention by 14.6 mg/dl in the SMS group and 12.3 mg/dl in the group training. Both were statistically significant (*p*-value: 0.001 and *P*-value: < 0.001 respectively). FBS value increase in control group after 3 months by 5 mg/dl (*P*-value =0.2). The calculated effect size in the group-based education and SMS groups were around 0.5, which means a moderate effect size.

**Table 2:** Comparison between means of FBS, 2 hr. pp. BS, HbA1c, and DSMQ sum score before and after study and between 3 study groups

**Variable**		**Control group** **Mean (S.D.)**	**SMS group** **Mean (S.D.)**	**Group-based education** **Mean (S.D.)**	**F**	**df**_**within**_	***P*-value**^**6**^
**FBS**^1^** (mg/dl)**	**before**	155.6 (62.9)	146.2 (50.5)	134.7 (41.3)	2	143	0.146^6^
	**after**	160.6 ± ( 52)	131.6 ± (28)	122.4 ± (28)	13.6	143	<0.001
	***p*-value**^**5**^	0.220^5^	0.001	<0.001			
	**difference**	5 (27)	-14.6 (29)	-12.3 (22)	8	143	0.001
**Effect size (Cohen's d)**^**7**^		0.18	0.5	0.56			
**HbA1c**^2^ **(%)**	**before**	7.7(1)	8(1.6)	7.9(1)	1	143	0.381
	**after**	7.9 ± (1.3)	7.7 (1.4)	7.4 (1)	2.1	143	0.12
	***P*-value**^**5**^	0.113	<0.001	<0.001			
	**Difference**	0.13 (0.6)	-0.36 (0.6)	-0.55 (0.45)	20	143	<0.001
**Effect size (Cohen's d)**^**7**^		0.2	0.6	1.2			
**2 hr pp BS**^3^ **(mg/dl)**	**before**	209.2 (11.9)	209(10.2)	179.2(8.2)	2.9	143	0.056
	**after**	209.3 (61.2)	172.8 (38)	160.6 (37)	13	143	<0.001
	***p*-value**^**5**^	0.770	<0.001	<0.001			
	**Difference**	2 (44)	-36.2 (47)	-18.5 (32.7)	8	143	<0.001
**Effect size (Cohen's d)**^**7**^		0.04	0.814.6	0.57			
**DSMQ sum score**^4^	**before**	6.6 (1.2)	6.2(1.3)	6.6(1.3)	0.8	141	0.461
	**after**	6.7 (1.1)	8 (0.9)	8.15 (0.7)	33.8	133	<0.001
	***P*-value**^**5**^	0.423	<0.001	<0.001			
	**Difference**	0.09(0.7)	1.7 (0.9)	1.4 (1)	43.8	132	<0.001
**Effect size (Cohen's d)**^**7**^		0.13	2.0	1.4			

^1^*FBS* Fasting Blood Sugar

^2^*HbA1c* Hemoglobin A1c

^3^2hr pp. BS: 2 hour post prandial blood sugar

^4^*DSMQ* Diabetes self-management Questionnaire

^5^calculated *P*-value from paired T test

^6^calculated *P*-value from Anova test

^7^Effect size for pair t. test (Cohen’s d) = $\frac{\left|{m}_1-{m}_2\right|}{sd}$

Also, 2hrPP BS decreased by 36.2 mg / dl in the SMS group (*P* value: < 0.001) and 18.5 mg / dl in the group-based education (*P* value: < 0.001), but increased by 2 mg / dl in the control group (*P*-value =0.8). The effect size of 2hrPP B.S. is high in the SMS group and moderate in the group training arm.

HbA1c value decreased by 36% in the SMS group (*P*-value: < 0.001) and 55% in the group- based education(*P*-value: < 0.001), but increased by 13% in the control group (*P*-value =0.1). The effect size is moderate in the SMS group and high in group-based education.

DSMQ sum score increased by 1.7 in the SMS group (*P*-value: < 0.001), 1.4 in the group- based education (*P* value: < 0.001), and 0.09 in the control group (*P*-value =0.4).

The effect size was large regarding DSMQ sum score in both group- and SMS-based education groups.

### Comparing the changes in glycemic level in 3 studied groups

The changes in glycemic level and DSMQ score showed statistically significant differences between the three groups (Table 2).

The comparison of fasting blood glucose changes between the three groups is statistically significant (F (2,143): 8 *P*-value: 0.001). The post hoc (LSD) test results showed significant differences between the control group and the group training or SMS groups. The difference between group-based education and the SMS groups was not statistically significant (Table 3).

**Table 3:** Pairwise comparison between studied groups (post hoc test: LSD)

	Control group Means diff (S.E.)	Group-based education Means diff (S.E.)
**Comparing glucose indices changes (Δ mean)**		
**FBS (mg/dl)**		
Group-based education	17.3 (5.3) *	
SMS group	19.6 (5.3) *	−2.3 (5.2)
**HbA1c (%)**		
Group-based education	0.7 (0.11) *	
SMS group	0.5 (0.11) *	0.2 (0.1)
**2hrPP BS (mg/dl)**		
Group-based education	16.6 (8.5)	
SMS group	34.3 (8.5) *	17.7 (8.3)*
**Comparison of DSMQ subscales scores after education**		
**Glucose management subscale**		
Group-based education	−1.4 (0.3) *	
SMS group	−1.5 (0.3) *	0.19 (0.25)
**Dietary control subscale**		
Group-based education	−2 (0.3) *	
SMS group	−1.9 (0.3*	−0.2 (0.25)
**Physical activity subscale**		
Group-based education	−2 (0.3) *	
SMS group	−1.6 (0.3) *	−0.3 (0.3)
**Healthcare use subscale**		
Group-based education	−0.8 (0.2) *	
SMS group	−0.37 (0.24)	0.42 (0.23)
**Total score**		
Group-based education	−1.5 (0.2) *	
SMS group	−1.3 (0.2) *	0.18 (0.2)

*FBS* Fasting blood sugar, *HbA1c* Hemoglobin A1c, *2hrpp BS* 2 hour postprandial blood sugar, *DSMQ* Diabetes Self-Management Questionnaire

*: *P* value< 0.05

Mean diff.: Δ mean values of the group in the column – Δ mean values of the group in the row (Δ mean values: mean of values after intervention- mean of values before intervention)

The result was the same in terms of HbA1c changes. This value showed a significant difference between the three groups (F (2,143): 20 *P*-value: < 0.001), and the result of the post hoc test showed that the result was unfavorable in the control group compared to the other two groups (Table 3).

Mean changes of 2 hr. PP-BS were also significantly different in 3 groups (F (2,143): 8 *P*-value: < 0.001). The post hoc test indicated a significant difference between control and SMS groups and between group-based education and the SMS groups. The SMS-based education improved 2 hr. PP-BS than group-based education (*P*-value < 0.035) (Table 3).

### Comparison of DSMQ scores between the three studied groups

Despite the lack of initial differences in subscales of the DSMQ questionnaire at the beginning of the study, all subscales and total scores were significantly different in the three groups after the study (Table 4).

**Table 4:** Comparison of means of DSMQ subscale and total score after the study between three study groups

	F	df _**within**_	***P***-value
**DSMQ total score**	33.8	133	< 0.001
**Glucose management subscale**	20.2	136	< 0.001
**Dietary control subscale**	27.4	138	< 0.001
**Physical activity subscale**	23.8	137	< 0.001
**Healthcare use subscale**	5.5	139	0.005

There was a significant difference between the control group and both group-based education and the SMS groups in terms of all subscales, except for the health care use subscale, in which no significant difference was detected between the control group and the SMS group (*P*-value = 0.8) (Table 4).

The group- and SMS-based education showed no significant difference in DSMQ total or subscales scores.

### Comparing total direct financial costs in group-based education and SMS-based education

The average direct financial cost for group-based education and SMS groups was roughly estimated and illustrated in Table 5. The cost is much higher in the group-based education than in the SMS group for patients and the organization. The total cost will be much higher for patients if we consider the time patients must spend attending group training sessions.

**Table 5:** Comparing the total direct financial cost spent for education in 2 educational group

	SMS Group		Group-based education	
	Calculation	Cost (IRR)	Calculation	Cost (IRR)
**The cost paid by the project implementing organization**				
**Cost of education tools**	270 IRR (Average fee for sending each SMS) * 90 (total number of SMS in three months)* 56 (number of participants)	1,360,800	0	
**Personnel wages**	150,000 IRR (staff salaries per hour) * 2 hours (spent for content selection and preparation in the form of short text messages) + 4 hours (1 hour/week spent replying to patient questions)	900,000	150,000 IRR (staff salaries per hour) * 20 hours (Spent for preparing PowerPoint, preparing, and holding a class)	3,000,000
**Total**		2,260,800		3,000,000
**The cost paid by the patient**				
**Out-of-pocket cost by patient**	0	0	20,000 IRR (Average cost of transportation by taxi) * 4 (class sessions)	80,000
**Total for all patients**	0	0	80,000 IRR * 56 participants	4,480,000

*IRR* Iranian Rial (the currency of Iran) 1 Iranian Rial (IRR) = 0.000004$

## Discussion

This study aimed to compare the effect of group-based education with SMS-based education and the control group on the glycemic level and the patients’ self-care. After 3 months of education, SMS- and group-based education have led to moderate to high effects on blood sugar indices and diabetes management behavior. Also, the results indicated that the DSMQ score and the changes in glycemic level are significantly different between the three groups after 3 months of education. The post hoc (LSD) test showed significant superiority of education by group-based education and SMS groups over routine follow-up (control group) in terms of FBS and HbA1c control and most DSMQ subscales. There was no significant difference between the control group and group-based education regarding 2 hr. PP BS. The differences between SMS and group-based education were not statistically significant in blood sugar indices except for 2 hr. PP BS, in which the SMS group achieved better lab data results. The overall direct cost of SMS-based education is much lower than that of group-based education.

These results were somewhat compatible with the previous study. A study by Rahnavard et al. compared three groups: group-based education, mobile education, and the control group. The intervention groups, either mobile or group training, had a higher self-care behavior score than the control group (*p* = 0.001). Except for self-care training in foot care, where the effect of mobile was more than the group-based education, there was no significant difference in other subscales. Likewise, in our study, SMS education was similar to group-based education except for the healthcare subscale. This result indicates that education via SMS is somewhat as effective as group-based education in diabetes self-care education [16].

In this regard, the cost spent for each training method is also essential. A study by Cobos-Campos et al. has shown that sending motivational text messages to quit smoking has been cost-effective [17]. Although our study did not evaluate the cost-effectiveness in detail, the overall costs incurred by the group-based and SMS- based education were compared. The result indicated that SMS-based education imposes a lower price on the health system and patients. Both methods demonstrated similar effects on patients’ blood sugar indices and self-care scores, so SMS-based education seems to be the preferred method.

One of the most important aspects of training is finding the right and effective frequency for sending messages via SMS and the duration of education. In different studies, sending text messages with different frequencies and duration has different results. In an investigation by Shetty et al., it has been shown that messages sent every 3 days for a year could not significantly reduce the mean hemoglobin A1c level. However, there was an increase in the percentage of people with HbA1c < 8% [18]. These results are inconsistent with the present study in which the hemoglobin A1c decreased significantly in the SMS group. This may be due to the longer duration of the intervention (1 year) than the present study (3 months). It is possible that the longer duration reduced the patients’ interest and, subsequently, the effect of the SMS messages. On the other hand, a longer interval between massages (every 3days) than in the present study (daily) could be the other cause of inconsistent results.

In a study conducted in Egypt, 34 people in the intervention group and 39 people in the control group were compared for 12 weeks. The intervention group received daily text messages on different aspects of diabetes education. The results showed that the mean HbA1c did not differ significantly between the two groups (*P*-value = 0.4). Still, the intervention group obtained a better score in the subscales of awareness, the importance of treatment, drug use, and self-care [19]. Perhaps the lack of difference in HbA1c in this study was due to the small number of participants. Also, the method of education can be effective in this regard. In our study, patients were asked to report their problems and questions about the disease weekly by SMS, and the experts answered their questions. They were also asked to respond to text messages daily. In the previous study, many incentives like free required drugs and a free visit by doctors were considered for participants. Our research was done in the field of the family physician, which always visits patients for free, so it cannot be considered an incentive. Therefore, it seems that the learner’s active participation in education can be more effective than an incentive. Also, perhaps the difference in people’s backgrounds about the disease and the desire to control it in different societies and countries is the reason for the differences in blood sugar control.

In a study by Dobson et al., people in the intervention group received SMS training in addition to routine care for 9 months, and the control group received only standard care. In this study, the participant determined the frequency of sending text messages, and the text messages were created according to the individual’s situation and were sent individually. This study showed a significant decrease in hemoglobin A1c in the intervention group after 9 months compared to the control group. A reduction in hemoglobin A1c has also been demonstrated in the present study, in which public text messages were sent. Therefore, although the time of this study was longer than the present study, it seems that based on the present study, sending public text messages can also be effective in controlling diabetes. So, it can eliminate the need to send private text messages, which requires spending more time and money [20].

In another study, 112 diabetic patients were divided into three groups control group, group training, and group counseling. The intervention showed that self-care in the counseling and training groups was better than in the control group, but the two groups were not significantly different (*P*-value = 0.394). In our study, self-care in group training was better than in the control group, but it was not different from the SMS training group. According to these two studies, it seems that the types of education on a small scale may not make any difference between the groups in terms of self-care behavior [21]. This difference may be significant in the larger study. Patient acceptance is a critical aspect of the educational program. Although we did not measure it by any scale, the patients’ feedback indicated that the method of teaching via SMS was more acceptable. It is necessary to measure this acceptance scientifically in future studies.

One of the strengths of the present study was the sampling method in which recruitment was performed from the patients under the coverage of three different family physician clinics in three different areas of the city, which allowed people with diverse socioeconomic characteristics to participate in the study. The overall sample can represent almost the entire urban community. Similarly, implementing all education by one educator in group-based and mobile education has reduced the bias.

One of the limitations of this study was the duration of intervention. Although the duration of 3 months was acceptable compared to other studies, increasing the time may have different effects on the outcome. Also, the study’s accuracy could be improved if more participants were employed. On the other hand, measuring participants’ acceptance of educational methods is reasonable, which has been neglected in our study. It is recommended to measure the acceptance in future studies. Measuring and comparing the sustainability of these two different education methods is also necessary.

It is recommended to compare the efficacy of sending text messages with different intervals in future studies and show which frequency has a more favorable effect.

## Conclusion

The present study showed that glycemic levels and self-care in type 2 diabetes could be better controlled in group-based or SMS-based education than in the control group. Comparing the two types of education via SMS and group training did not show significant differences in diabetes control except for mean blood sugar 2 hours after the meal, which was better controlled in the SMS group. Due to spending a lot of time and money on group-based education, it seems better to educate this group of patients via SMS instead of traditional group-based methods whenever possible.

### Clinical relevance statement

Education by mobile SMS had an equal or even slightly better effect on the management of diabetes than the costly method of group-based education, which can be of particular importance, especially in a developing country with limitations on resources like the environment in which the study was conducted. The SMS-based education showed clinically moderate to high effects on controlling glycemic level and diabetes management behavior. In addition, this method was more effective in diabetes management than routine face-to-face education by healthcare workers.

## Data Availability

The datasets used and/or analyzed during the current study are available from the corresponding author on reasonable request.
